# The Role of Master Regulators in the Metabolic/Transcriptional Coupling in Breast Carcinomas

**DOI:** 10.1371/journal.pone.0042678

**Published:** 2012-08-27

**Authors:** Karol Baca-López, Miguel Mayorga, Alfredo Hidalgo-Miranda, Nora Gutiérrez-Nájera, Enrique Hernández-Lemus

**Affiliations:** 1 Computational Genomics Department, National Institute of Genomic Medicine, México City, México; 2 School of Sciences, Autonomous University of the State of México, Toluca, México; 3 Cancer Genomics Laboratory, National Institute of Genomic Medicine, México City, México; 4 Proteomics Core Facility, National Institute of Genomic Medicine, México City, México; 5 Center for Complexity Sciences, National Autonomous University of México, México City, México; Università del Piemonte Orientale, Italy

## Abstract

Metabolic transformations have been reported as involved in neoplasms survival. This suggests a role of metabolic pathways as potential cancer pharmacological targets. Modulating tumor's energy production pathways may become a substantial research area for cancer treatment. The significant role of metabolic deregulation as inducing transcriptional instabilities and consequently whole-system failure, is thus of foremost importance. By using a data integration approach that combines experimental evidence for high-throughput genome wide gene expression, a non-equilibrium thermodynamics analysis, nonlinear correlation networks as well as database mining, we were able to outline the role that transcription factors MEF2C and MNDA may have as main master regulators in primary breast cancer phenomenology, as well as the possible interrelationship between malignancy and metabolic dysfunction. The present findings are supported by the analysis of 1191 whole genome gene expression experiments, as well as probabilistic inference of gene regulatory networks, and non-equilibrium thermodynamics of such data. Other evidence sources include pathway enrichment and gene set enrichment analyses, as well as motif comparison with a comprehensive gene regulatory network (of homologue genes) in Arabidopsis thaliana. Our key finding is that the non-equilibrium free energies provide a realistic description of transcription factor activation that when supplemented with gene regulatory networks made us able to find deregulated pathways. These analyses also suggest a novel potential role of transcription factor energetics at the onset of primary tumor development. Results are important in the molecular systems biology of cancer field, since deregulation and coupling mechanisms between metabolic activity and transcriptional regulation can be better understood by taking into account the way that master regulators respond to physicochemical constraints imposed by different phenotypic conditions.

## Introduction

It is known that tumors could depend on energy production pathways that are different from those of normal cells. These unique pathways require in some cases the expression and function of so-called *tumor-specific enzymes*. Some of these glycolytic enzymes, as well as other modulators of tumor behavior, have recently been analyzed in search for a clue that inhibition of such enzymes or appropriate tuning of such modulators should deprive tumors of energy, while leaving non-transformed cells unaffected. Recent findings seem to point out to several so-called *metabolic transformations* that permit neoplasms survival, thus suggesting a role of metabolic pathways as potential pharmacological targets [1]. In fact, preliminary experiments on animals with hepatocellular carcinoma have indeed shown very encouraging results. It appears that modulating the energy production pathways of tumors is poised to become a substantial research area for cancer treatment [2]. The role of perturbed local cell energetics in association with cancer is not new. In the past, under several instances, relationships seem to appear between metabolic variation and tumorigenesis, spread and dissemination of malignancy. In recent times a growing interest (or best a revival of it) has taken place and evidence seem to suggest closer connections than those suspected. For instance, the importance of glycolysis in cancer development [3]. It has been discussed how a combination of agents that inhibit both energy production and cell signaling may provide a novel and effective approach to target pancreatic cancer effectively.

Thermodynamic studies at the transcriptional [4], epigenetic [5], [6], and metabolic [7] levels have pointed out to energetics as playing a non-trivial role in the onset and development of malignancy. In the particular case of this paper, we will focus on the relationship between transcriptional de-regulation of a set of genes that present transcription factor (TF) and metabolic activity (some of them) while at the same time have been associated with the presence of breast cancer. We will then study its regulatory and thermodynamical behavior by means of gene expression data obtained from genome-wide analysis experiments in RNA from biopsy-captured tissue of both primary breast cancer and normal breast.

The role of gene interaction networks have also been extensively mentioned in relation to cancer phenomenology, it has been claimed that these network effects are, in fact much more important than individual gene contributions [8]. Some of these networks are indeed related to energetic and metabolic processes [9], tyrosine-related deregulation [10], and immunity weakening [11]. One usually think of tumor cells as having successful mechanisms to evade normal control and cell regulation of proliferation and apoptosis. Alterations in gene expression have become a better (but far from completely) understood component of normal development and disease progression. In particular, TFs have become a promising target for therapy. In brief, gross alterations in TF regulation would result in cascade triggering affecting both the whole cell cycle and the metabolic activity thus resulting in possible development of cancer. Many people have come to conclude that *cancer* is a *transcriptional disorder* disease [12]–[15], while, as we have mentioned other authors have recently turned their attention to the metabolic and energetic component [7], [9], hence a possible connection between these two approaches could be found in the *energetic deregulation* 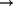 *transcriptional disorder* leading both to cascade triggering and metabolic disorders related to neoplasm formation and development. For these reasons this paper will attempt to model the role of TFs at both the energetics (thermodynamic) level and the network approach.

## Analysis

**Figure 1:**
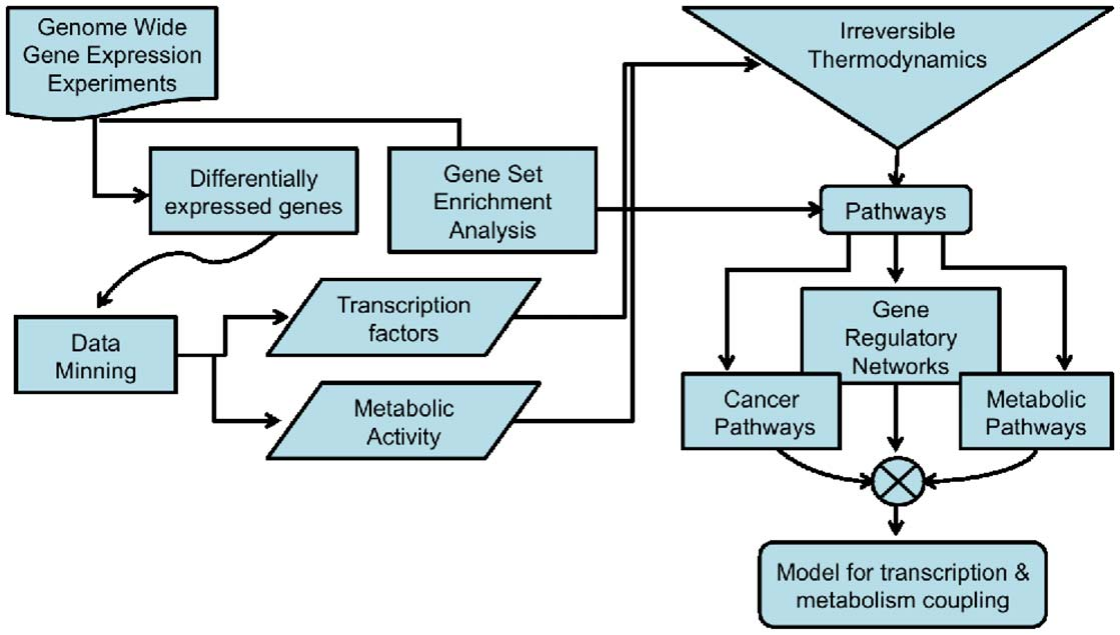
Flow chart design for this work. It is noticeable that this is an hybrid model which incorporates both data-driven discovery (gene expression analysis, gene-set enrichment and probabilistic network inference) and hypothesis driven inquiries (data mining and non-equilibrium thermodynamics modeling).

**Figure 2:**
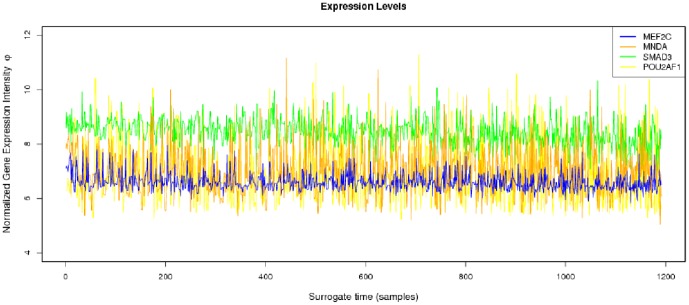
Gene Expression Intensity profile. We can notice strong stochasticity in the signals. However, a definite background tendency may be identified, as it may be clearer when examining the concentration profile.

**Figure 3:**
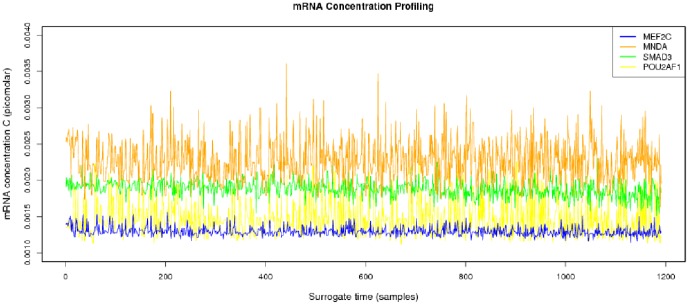
mRNA concentration profile. In spite of strong stochasticity, we can notice some trends. For instance, there are some genes with a low concentration and low variability while others present larger concentrations and variabilities.

**Figure 4:**
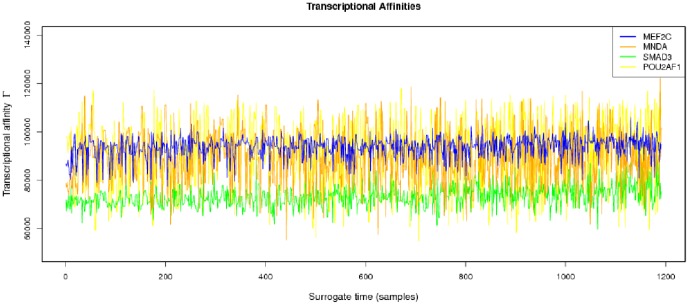
Transcriptional affinities profile , a strong stochastic behavior can be noted, however when considering the associated chemical potentials of transcription (see Fig. 5) definite trends arise.

**Figure 5:**
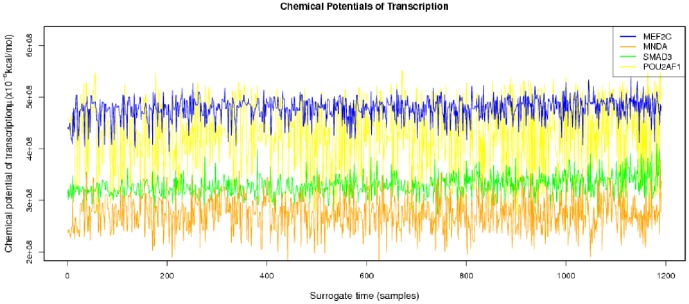
Chemical potentials of transcription profile. As in the case of the concentration profiles we can notice some trends in spite of stochasticity. There are some genes with a low concentration and low variability while others present larger concentrations and variabilities.

**Figure 6:**
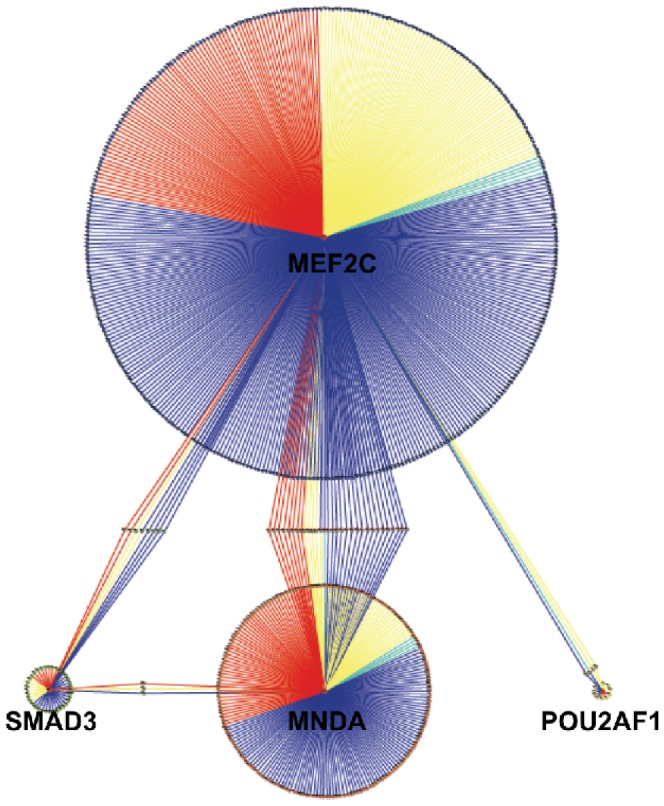
Inferred gene regulatory network [**10**], [**16**]. Links colored in red represent interactions associated (in the literature, from data mining) with breast cancer, yellow links are interactions associated with other types of cancer, turquoise links are interactions associated with metabolic disorders and navy blue links are otherwise. We could notice that some genes are regulated by more than one of these transcription factors as is the case with RAD52, ADH1C, OIP5, ELK4, PEX10, GAA, FTHP1 and ADAP1 which are co-regulated by MEF2C and SMAD3; of STMN1, STAU1 and the c10ORF10 transcript which are co-regulated by SMAD3 and MNDA; of CSH1, FANCI, FHIT, CDKN2A, PRC1, CENPF, MAP3K1, BNIP3, HCLS1, TAF15, PCBP2, SPAI7, SLC38A1, PASK, HIST1H2BD, POLR2I, UPK3B, EHD1, PRICKLE3, LOC91316, ANAPC2, LST1, RRP12, c9ORF6, BRP44, CLEC4A, TMEM194A, TPO3B, HIST1H2AM and ZFP37 which are co-regulated by MEF2C and MNDA; and by c14ORF1, DOK5, AFF1, and CADM4 which are co-regulated by MEF2C and POU2AF1. In the case of these double-regulatory interactions there are some cases in which both regulatory interactions are associated with a certain phenotype and other cases in which different (or no) phenotype is associated for each link.

**Figure 7:**
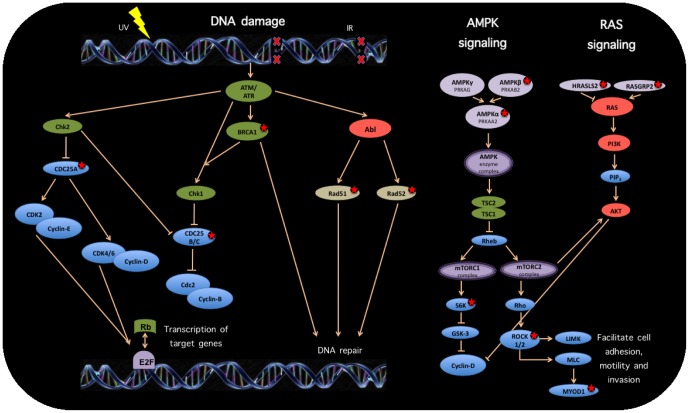
Cancer related deregulated pathways. Statistical enrichment of deregulated pathways within our differential gene-sets include canonical cancer pathways such as DNA damage repair, AMPK and RAS. Molecules marked with a red star correspond to differentially expressed genes in the cancer/control contrast.

**Figure 8:**
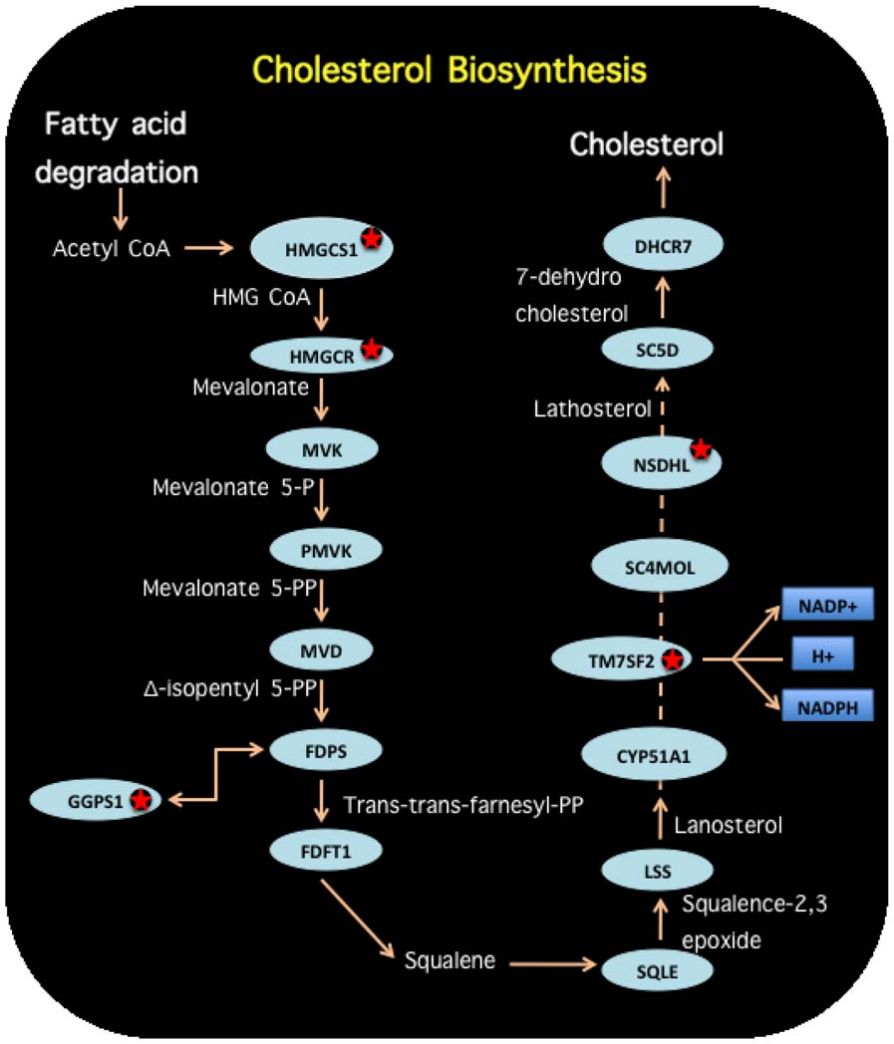
Metabolism related deregulated pathways. Statistical enrichment of deregulated pathways within our differential gene-sets include metabolic pathways. For instance, both branches of the cholesterol biosynthesis pathway are affected. Molecules marked with a red star correspond to differentially expressed genes in the cancer/control contrast.

One of the cornerstones of contemporary genomic studies, in particular of the systems biology approach, is data integration (DI). DI is useful to *make sense* out of the extremely large corpus of experimental evidence given, for instance by genome-wide expression analysis. With the continuous advent of novel techniques in high throughput molecular biology and the *‘omics* maybe just one thing has been established: Complex biological systems need to be studied from several standpoints to unveil the actual mechanisms behind them. In the present case, our aim is to sketch some hints for a proposal of functional mechanisms behind gene expression in cancer and cell energetics. The analysis work-flow for the present study was as follows (see also Figure 1):

1. Statistical pre-processing of the microarray gene expression data.
2. Determination of differentially expressed genes and statistical significance assessment.
3. Data mining for functional features within the statistically significant differential expression gene set.
4. Non-equilibrium thermodynamics calculations (Figures 2, 3, 4, 5).
5. Probabilistic inference of gene regulatory networks.
6. Pathway statistical enrichment analysis.
7. Search for common non-linear correlations found for human MEF2C in this work (Figure 6) that are present also in a highly curated A. Thaliana transcription factor database, indicating modular conservation among species.
8. Gene Set Enrichment Analysis applied to the 1191 samples expression matrix to look up for dysregulated functions and pathways as a complement for the gene analysis in cancer and metabolic pathways (Figure 7 and Figure 8).

### Differentially expressed genes

After pre-processing (background correction, normalization and summarization) of the samples [16] according to the RMA algorithm [17], we proceeded to implement a statistical analysis by using linear modeling (limma) to look up for significant differentially expressed genes (full data matrix available upon request). Empirical Bayes and other shrinkage methods are used to borrow information across genes making the analyses stable even for experiments with small number of arrays. This method allows very general experiments to be analyzed as easily as single replica experiments. The approach requires two matrices, the first one is called the *design matrix* which gives a representation of the different RNA targets which have been hybridized to the arrays. The second one, or *contrast matrix* which allows the coefficients defined by the design matrix to be combined into contrasts of interest. Each contrast corresponds to a comparison of interest between the RNA targets [18].

### Data mining for metabolic and transcription factor activity

Once we had a set of differentially expressed genes, we proceeded to implement a data mining search over it. Search parameters include the following constraints:

- Genes that are well known transcription factors, reported not only by sequence homology but also by actual experimental evidence.
- Genes that have been associated in the literature with the presence of breast cancer (higher scores) or any other tumors -liquid neoplasms were excluded- (lower scores).
- Genes whose protein products are related to cellular level metabolic pathways.
- Genes whose transcripts possess a complete physicochemical characterization, e.g. Affymetrix® calibration probes have reported free energies of formation.

From the set of genes included in the GeneChip® under study (namely Affymetrix HGU133-A) which were statistically significant in their differential expression between tumors and controls, we built sets that satisfy the aforementioned constraints. Then we made the intersection set of all these. This set, that we will call hereon a *Core set* consisted in four genes, namely *MNDA*, *POU2AF1*, *MEF2C* and *SMAD3*. In what follows we will analyze in detail the non-equilibrium thermodynamics of transcription as well as the regulatory network structure of such genes within a sample set of 1191 microarrays.

### Non-equilibrium thermodynamic model

On general grounds, the finding of relevant genes associated with a cancer phenotype is based on determining features such as differential expression patterns. However, two important issues that should be also taken into account besides the gene expression levels, are the energetics within the cell and the physicochemical properties of the biomolecules involved in transcriptional regulation (transcription factors in particular). Both features could be responsible for TF activity since they affect the mechanism behind the activation of target genes according to previous specific cellular energetic conditions.

An interesting trend in the transcriptional energetics in some well-studied genes [19] is that, the values of the activation energies are in general lower for genes that act as transcription factors and higher for genes with no-known TF-activity. The physicochemical meaning of this finding seem to point-out to transcription factors as genes whose expression is regulated by lower activation-energy barriers. Since TF's are involved in the transcriptional activation of other genes, it is expected that they are synthesized first when energy is started to being released by metabolic processes in the cell. Transcriptional targets should, in general be synthesized later and with higher activation energies. These higher saturation limits for the chemical potentials of TFs suggest both stability and spontaneity in the expression of these as compared to target genes.

In brief, since master TFs are needed in early stages of whole-genome transcription (i.e. *upstream* in the regulatory cascade) in order to kick-start such processes and, by taking into account that transcriptional regulation has been characterized as an activated process [20]; a hierarchy in the synthesis of mRNA templates (and, further on, in their product proteins) is established in terms of the corresponding activation energies -as given by their chemical potentials- and of the availability of free energy in the cellular environment. Then, by calculating thermodynamic properties for TFs, it is possible to unveil the order or priority in their activation which is dependent on energy accessibility.

A non-equilibrium thermodynamical theory of gene regulation has already been proposed [20]. As it was shown the thermodynamic analysis of transcriptional regulation presents several challenges, in particular associated with the fact that the cell is a *small* system, in the sense that the role of fluctuations and *noise* plays a rather fundamental role for its characterization. Systems outside the domain of the thermodynamic limit are characterized by large fluctuations and hence stochastic effects need to be taken into account. An extremely important conundrum in contemporary thermal physics lies in the connections between probability and thermodynamics. A developed theory exists however, called *mesoscopic nonequilibrium thermodynamics* (MNET) [21] which specifically addresses the issue by considering the stochastic nature of the time evolution of small non-equilibrium systems, in a context which is extremely close to our work. To account for stochasticity one needs to recognize that scaling down the description of a physical system brings up energy contributions that are commonly neglected in thermodynamical descriptions. The time-evolution of these systems could be described as a generalized diffusion process over a potential landscape in the space of mesoscopic variables. This process is driven by a generalized mesoscopic-thermodynamic force whose stochastic origin could be tracked back by means of, for example, a Fokker-Planck-like analysis [21]. These classes of formalisms are appropriated in the case of activated processes, for instance, a system crossing a potential barrier. The complex biochemical reactions involved in transcriptional regulation belong also to this category.

The present theoretical framework [20] shares similar ideas with MNET (although treated in a less formal way due to present unavailability of information regarding the non-local probability distributions) and deals with intensity levels of gene activity as measured in whole genome gene expression profiling on GeneChips. It is based on the thermodynamics of hybridization [22] that considers a basic two-state model that quantifies mRNA concentrations by competitive hybridization [23], [24]. This non-equilibrium thermodynamical theory has been used to study the role of transcription factors in the phenomena of anomalous transcriptional bursts [19], [25].

As is usual in non-equilibrium thermodynamics we will assume that a generalized entropy-like function 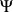 exists, which may be written in the form [26], [27]:
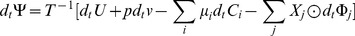
(1)
Eq. 1 is a formal extension of the Gibbs relation of equilibrium thermodynamics. The quantities appearing are as usual: 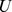 is the internal energy, 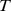 is the local temperature, 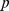 and 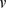 the pressure and volume, 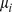 is the chemical potential for the 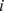-th mRNA species. 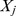 and 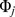 are extended thermodynamical fluxes and forces [26]. For a multicomponent mRNA mixture (under fixed volume and pressure), the set of relevant variables consists in the temperature 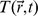 and concentration of each gene species 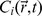 as the slow varying (classical) parameters set and the *mass flux* of these species 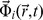 and their corresponding forces 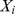 as fast variables. These latter variables will take into account the presence of inhomogeneous regions (concentration domains formed because of the gene regulatory interactions) to correct the predictions based on the local equilibrium hypothesis.

The non-equilibrium Gibbs free energy for a mixture of 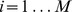, mRNA transcripts reads:
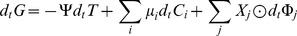
(2)Quantities are local fields defined as usual, (e.g. 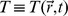), within the mentioned formalism one can consider that a generalized entropy-like function 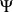 exists [26], [27], also T is the temperature, p the pressure, 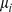 the chemical potential, etc.; 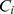 is the concentration for species 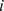, 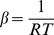, with 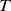 the absolute temperature and 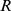 the gas constant, 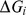 is the free energy of hybridization of 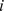, 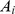 is a parameter that sets the scale of intensity [22] corresponding to the saturation limit 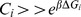 ; 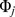 and 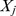 are extended thermodynamical fluxes and forces that take into account non-local effects.

If we recall from reference [20] given the relation between gene expression intensity 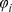 and concentration 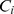, we have that:
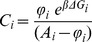
(3)After this, a proposal on the form for the extended fluxes and forces should be given. Hence, we are proposing a system of linear (in the forces) coupled fluxes with memory [20].

The constitutive equations are,
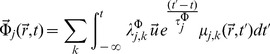
(4)

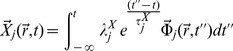
(5)The 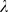
's are time-independent amplitudes, 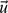 is a unit vector in the direction of mass flow (the nature of 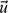 will not affect the rest of our description, since we will be dealing with the magnitude of the mass flux 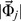) and 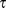
's are relaxation times considered path-independent scalars. Since we have a linear relation between thermodynamic fluxes and forces some features of the Onsager-Casimir formalism will still hold.

Irreversible coupling is given by Eq. 4 and 5, nevertheless due to the fact that actual transcription measurement experiments are made either on homeostasis (steady state) settings or within time series designs with intervals several orders of magnitude larger than the associated relaxation times (which are of the order of a few molecular collision times) it is possible to take the limits 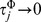 and 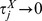, then the integrals become evaluated delta functions to give:
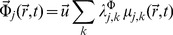
(6)

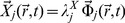
(7)Also due to the spatial nature of the experimental measurements (either RNA blots or DNA/RNA chips measure space-averaged mRNA concentrations) it is possible to work with the related scalar quantities instead, to give:
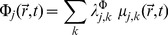
(8)

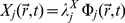
(9)Substituting Eq. 8 and 9 into Eq. 2 one gets:
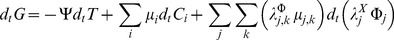
(10)If we assume that the generalized transport coefficient 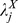 is independent of the flux 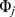 we can write:
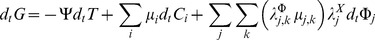
(11)Or in terms of the transcription regulation *chemical potentials* 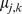


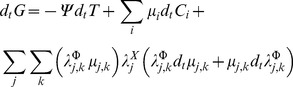
(12)In the constant transport coefficient approximation, Eq. 12 reads:
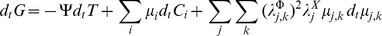
(13)Defining generalized transport coefficients 
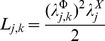
.

If we change variables in equation 2 from concentration to gene expression intensity (by using equation 3) and introduce the fluxes, forces and generalized transport coefficients, we could rephrase it as follows:
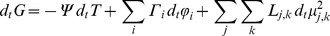
(14)The resulting *affinity of transcription* i.e. the thermodynamic *conjugate* variable to the probe intensity 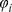 is given by:
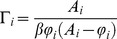
(15)The quantity 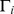 plays the role of a *chemical affinity* for the gene expression process. As gene expression is a process that follows thermal activation kinetics, 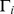 is a temperature dependent variable. This dependency shows up both explicitly (by the value 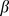) and also due to indirect temperature dependency given by the saturation constants 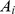. 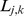 are coefficients related to gene cross-regulation and 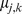 are the chemical potentials associated with transcriptional regulation [19], [20].

The related chemical potential of transcription [20] is given by:
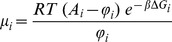
(16)Since we have reliable experimental data for the expression levels 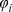 from 1191 whole-genome gene expression experiments, it is possible to calculate the gene transcriptional affinities and chemical potentials for the set of genes of interest from equations 15 and 16 (we also have good values for the constants 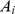 taken from *spike-in* experimental data provided by the gene-chip manufacturer). Since these experiments have been made without the use of any *knock-out* or *knock-down* techniques (i.e. all genes are subject to their corresponding regulatory interactions), the gene expression levels 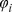 used to calculate the chemical potentials and transcriptional affinities have already incorporated (although in an implicit way) the effect of transcriptional regulation as given by the gene regulatory mechanisms depicted in the third term at the r.h.s. of equation 14.

### Non-linear correlation inference of regulatory networks

To deconvolute a Gene Regulatory Network (GRN) related to primary breast cancer we applied a methodology based on a local pattern-sharing measure as surrogate to actual gene-gene interactions to our dataset(see Materials and Methods - 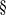 Experimental datasets). The goal of deconvolution methods is inferring GRNs based on statistical dependencies within the joint probability distribution of gene expression for all genes within a given gene set. Typical means to reach this goal consist in the quantification of the new information content that arise when we look at the full joint probability distribution when compared to a series of successive independence approximations. According with the method given in reference [10], we calculated measures of non-linear correlation between the normalized expression values (22238 probesets) and the core set of 4 genes. Threshold-analysis was made on such measures to look up for *statistical significance* in the inference and based on their IBS index value we selected a set of 712 genes. The optimal network (within the given approximations) was found by a Maximum Entropy Method as is shown elsewhere [10] and was validated by several (mostly *in silico* and *database-mining*) methods [16].

### Biochemical pathway statistical enrichment analysis

Reactome [28] biological/biochemical pathway over-representation analysis was performed to determine the Reactome pathways in which gene IDs in our list were strongly enriched. Reactome is a an open-source, open access, manually curated and peer-reviewed pathway database that may help to understand the biological context of genomic data. Significance assessment was also made by means of ‘urn model’ hypergeometric distribution tests.

## Materials and Methods

### Experimental datasets

A curated set of 1191 whole genome gene expression profiles was generated [16] from datasets for several publicly available experiments deposited in the GEO database [29]. These experiments were performed in total mRNA extracted under the GPL96 protocol [30] which is based on the Affymetrix HGU133A microarray GeneChip platform. In the case of experiments including some kind of treatment or cell modification we only took the unaltered samples to include them in our analyses. Details are given in Table 1. Further information is available in the corresponding GEO entries and/or may be available upon request. In the case of human mRNA samples taken directly from organ tissue (by a biopsy) and not from cultured cell-lines, it is extremely difficult to design time-course experiments. Thus, in order to study a surrogate model of transcriptional de-regulation, we proposed the following alternative to look for correlations: After quality control pre-processing, background correction and normalization of the microarrays, the samples were prioritized (ordered) according to their *BNIP3* (Affymetrix-probe ID 201848_s_at) expression level. *BNIP3* is a well known marker of progression and malignancy in primary breast cancer that correlates both with lab tests and clinical trials [31]. By ordering the independent, steady-state samples in this manner it is now possible to look up for correlation patterns of gene expression.

**Table 1:** GEO [29] identifier and references for the Microarray experiments used here, first column is GEO key ID, second and third columns are the corresponding number of samples cases/controls resp.

GEO ID Series	Tumors	Controls	Reference
GSE1456	159		[68]
GSE4922	249		[69]
GSE7390	198		[70]
GSE2603	99		[71]
GSE2990	125		[72]
GSE3494	251		[73]
GSE1561	49		[74]
GSE15852		43	[75]
GSE9574		15	[76]
GSE6883		3	[77]

Fourth column is the reference entry.

### Statistical and Computational tools

Microarray pre-processing of the data was performed by using the affy library in BioConductor running under [R] on a 128 Gb RAM 8-Power5+ dual core-processor, symmetric multiprocessing (SMP) unit by IBM. Whereas all statistical tests were performed on a Dell Precision Series 8 Gb RAM QuadCore Workstation by using limma package in [R]/BioConductor. Information theoretical measures (Information Based Similarity) were calculated with the ibs program. Such information theoretical measures were used to infer regulatory interactions between TFs and target genes, i.e. to deconvolute the associated gene regulatory network [10], [16]. As is thoroughly discussed in reference [10], genes highly correlated in their expression patterns are likely to be transcriptional partners. Since dynamic correlations between genes and their TFs are of a nonlinear character, we have used mutual-information related measures instead of linear measures such as Pearson's correlations and covariances. Graphical depiction and network analyses were performed with Cytoscape. Non-equilibrium thermodynamics calculations and other analyses were performed with custom [R] and shell scripts. Pathway enrichment analysis was made by means of hypergeometric testing of databases by Reactome [28]. Gene Set Enrichment Analyses were performed with the GSEA Java library [32].

## Results

### Core regulation genes

From the set of differentially expressed genes, data mining techniques were implemented to determine a set of genes that at the same time were involved in metabolic activity at the cell level and/or in cancer; and possess experimental data to accurately determine the parameters involved in our non-equilibrium thermodynamic model. We have then come to investigate cell energetics in relation with mRNA expression of the following TFs: *MNDA*, *POU2AF1*, *MEF2C* and *SMAD3*.

*MNDA* acts as a transcriptional activator/repressor in the myeloid lineage [33]. Also plays a role in the granulocyte/monocyte cell-specific response to interferon and stimulates the DNA binding of the transcriptional repressor protein *YY1* [34]. It belongs to a family of P200 proteins that inhibit cell cycle progression and modulates cell survival. *POU2AF1* is a transcriptional coactivator [35]–[37] that specifically associates with either OCT1 or OCT2. It is located in a so-call *Tumor suppressor region* in 11q22-23 and it is suspected to modify non-coding effects on gene expression [35]. *POU2AF1* amplification has been detected in multiple myeloma cells and this copy number variation also reflected in over-expression at both the mRNA and protein levels [37]. *MEF2C* is a transcription activator which binds specifically to the MEF2 element present in the regulatory regions of many muscle-specific genes. In fact, *p38*, *ERK5*, and *MEF2C* itself have been recently described as novel downstream Brk (*PTK6*) effector pathways [38] supposedly playing a role in primary breast cancer. The actual mechanism seems to be related with ERK5 being an input to cyclin D1 transcriptional up-regulation, maybe following MEF2C-dependent up-regulation and recruitment of *c-JUN* to the cyclin D1 promoter [38]. *MEF2C* is a transcriptional enhancer whose biological function in human breast cancer is still unknown. However, it has been shown that its chromosomal localization is assigned to the so-called *mammary cancer susceptibility 1* locus (Mcs1) on chromosome 2q1 segregating with the sensitivity to mammary cancer development in a murine model [39]. *SMAD3* is a transcriptional modulator activated by *TGF-* 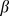 (transforming growth factor) and activin type 1 receptor kinase. *TGF-* 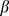 induces a cytostatic response in most normal cell types, but in cancer cells promotes metastasis, and its high expression is correlated with poor prognosis [40]. Knocking-off experiments have showed that the *TGF-* 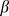-induced SMAD3-mediated transcriptional response, was mitigated and enhanced by *SMAD3* and *SMAD2* knockdown, respectively, and this could be directly correlated with divergence in the regulation of tumor angiogenesis in vivo [41].

In view of the importance of these genes in the onset and development of breast cancer, we have decided to investigate both the energetics and connectivity of their functions in both normal and neoplastic cells. We will discuss the role of TF activity, activation energies and chemical potentials of transcription as outlined and we will suggest some routes to follow to further understand the role of metabolic changes (both at energetics and pathways level) in breast malignancy.

### Thermodynamic analysis of *MNDA*, *POU2AF1*, *MEF2C* and *SMAD3* transcription factors

The role of integrative analysis in modern (high throughput) genomics is to present a basis for hypotheses generation that may be tested in more specific and detailed studies. In this sense our non-equilibrium thermodynamics calculations (plus some assumptions regarding energy release within the cell) supply a means to try to unveil causal structure of the regulatory interactions from correlation analysis (such as the network study presented here), thus providing a more appropriate frame for study.

The parameters needed to calculate the intensity-dependent concentration, the expression affinity and the chemical potential of transcription for *MNDA*, *POU2AF1*, *MEF2C* and *SMAD3* are shown in Table 2 and the explicit calculations are in Table 3. If we look at the gene expression profiles in the surrogate model (Figure 2), we notice the presence of stochastic components with a high variance. In the mRNA concentration representation (Figure 3) although we retain stochastic evolution, it is easier to notice differences between the concentrations for the considered transcripts. Lowest concentration values of *MEF2C* are present in almost all stages of tumor progression. This gene also showed the lowest variance between sampling points as it could be seen in figure 3. Even if *POU2AF1* expression levels were almost as low as those of *MEF2C*, its variance was much greater and in some instances mRNA concentrations doubling its baseline level are present. *SMAD3* was in general found to present medium expression levels and medium variance. *MNDA* showed the highest concentrations (about 3.5 times that of *MEF2C* in some instances) and also showed the greater variability. With regards to transcriptional affinities (Figure 4) similar comments can be made as with expression levels (these are after all *thermodynamic conjugate variables*). We can however notice higher variability around the mean behavior which points out to the possible presence of *activation processes* taking place. If we consider that transcriptional affinity and transcription level are conjugate (i.e. its product is an *energy* term), the fact that affinities (for example in the case of *MNDA*) present a pattern of variance different from that of the expression levels, imply the existence of *energy fluctuations* that may be due to activation processes. This assertion becomes clearer when we consider the time evolution of the chemical potential (Figure 5). The chemical potential associated with *MNDA* transcription presents the lowest values (thus it is easier to synthesize because of its lower activation energy barrier) as well as the higher variance. *SMAD3* also presented relatively low chemical potentials and medium values of variance. In accordance with mRNA concentration profiles, *POU2AF1* showed medium chemical potentials but large variance and *MEF2C* showed the largest mean values of chemical potentials but a lower variability.

**Table 2:** Thermodynamic parameters needed for the calculations of 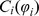, 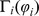 and 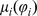 at *physiological temperature* (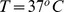 or, 310.15 K).

Gene	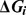 (kcal/mol)	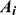	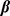 (mol/kcal)
*MNDA*	433.97	3105	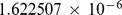
*POU2AF1*	473.5	4684	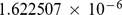
*MEF2C*	472.81	5110	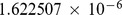
*SMAD3*	465.08	4497	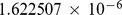

**Table 3:** Thermodynamic calculations of 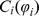, 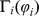 and 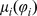 at *physiological temperature* (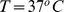 or, 310.15 K).

Gene	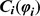	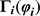	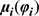
*MNDA*	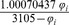	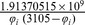	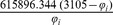
*POU2AF1*	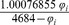	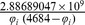	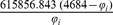
*MEF2C*	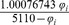	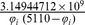	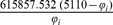
*SMAD3*	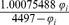	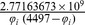	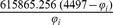

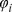
 is the corresponding gene expression intensity value, concentration [ = ] picomolar, chemical potentials [ = ] 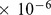 kcal/mol.

### Non-linear correlation networks

The results of the inferred non-linear correlation network centered in the transcriptional regulation partners for the *core regulation genes* could be seen in Figure 6. Indirect correlations between these 4 genes were not pruned (e.g. by using the Data Processing Inequality [42]) since it is precisely through these links (and their corresponding regulatory and signalling pathways) that the interconnection between metabolism and transcriptional regulation is more clear, as it may be evident later. Referring to Figure 6, it may be noticed that genes participating in the interactions represented by links colored in red have been related in the literature with breast cancer, whereas yellow links are associated with other types of cancer-related genes; turquoise links are interactions associated with genes in metabolic disorders and navy blue links are otherwise (complete list of references available upon request). Some genes are transcriptionally correlated with more than one of these transcription factors, as is it can be seen in the network and noticed in the caption of Figure 6. Hence, close to a half (about 45% indeed) of the regulatory interactions found in this network analysis have been reported to play a role either in cancer, metabolic deregulation or both.

The structure of the GRN (Figure 6) resembles a dual-control loop centered in core genes MEF2C and MNDA (and its targets-interactors) that may be fine-tuned by the action of POU2AF1, SMAD3 and its associated genes. The pathway analysis performed (see the following subsection) presents also indirect evidence pointing in this direction. However, conclusive assertions could only be made after more detailed and specific studies in regulatory dynamics are performed by means of actual time-course experiments.

The somewhat special role of MEF2C related interactions is worth-mentioning, in particular with regards to the regulatory effects of its MADS-box structure. It was previously shown that MADS-box gene transcription factor is a common regulator extremely conserved across plant and animal kingdoms. This is particularly the case in either *H. Sapiens* and *A. Thaliana*. In particular, from our core genes, human MEF2C belongs to the MADS-box genes family [43]. A representative short aminoacid sequence (60 aa) from MEF2C human protein (MEF2C_Hs) was taken from reference [43] and used for searching conserved domain sequences within A. Thaliana's MADs family members, by using NCBI Blast protein tool [44]. Protein conserved domains from Putative MADS-box family transcription and Mef2 myocyte enhancer factor 2 families sharing domain-architecture were found, 50 and 101 respectively (E-value 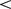
1E-07).

From the inferred gene regulatory network, MEF2C target genes: TAF12 and POLR2I correspond to be *Arabidopsis* homolog genes with TAF12 and NRPB9A/B. The one-to-one gene interaction in human between MEF2C and POLR2I founded in the present work corresponds to 25 MEF2C-MADS-box conserved domain genes and NRPB9A interactions as results from a search in the *Arabidopsis thaliana root transcriptional interactions database* [45].

### Pathway analysis

In order to sketch the role of specific biochemical sets of reactions related to energetic and transcriptional deregulation processes in the regulatory networks just referred, we performed pathway-related analyses in the list of 712 genes obtained from the IBS calculations. In particular, we made a pathway enrichment analysis for the statistical over-representation of pathways in this gene set.

Significantly enriched pathways include cancer related pathways such as *Activation of ATR in response to replication stress*, *Association of RAD51 with RAD52:DNA double-strand break ends*, as well as metabolic activity related pathways like *Cholesterol biosynthesis* and *Addition of galactose by beta 4-galactosyltransferases*. With regards to the activation of ATR in response to replication stress [46]; ATR belongs the PI3/PI4-kinase family, and is most closely related to ATM sharing similarity with *rad3*, a cell cycle checkpoint gene required for cell cycle arrest and DNA damage repair in response to DNA damage [47]. This kinase has been shown to phosphorylate checkpoint kinase CHK1, checkpoint proteins RAD17, and RAD9, as well as tumor suppressor protein BRCA1 [48]. Deregulation of the ATR signaling pathway has been related with various instances of breast cancer development [49]–[51]. Figure 7 shows some prototypical cancer pathways that are deregulated (abnormally expressed genes are marked with a red star) in the 1191 whole-genome gene expression experiments analyzed. Those genes are transcriptionally correlated with one or more of the genes in our *core* set as it could be verified in a closer analysis of the network depicted in Figure 6 (Cytoscape .cys file for Figure 6 available upon request). We can see that mRNA levels for CDC25A, CDC25C, BRCA1, PRKAB2, S6K, ROCK1 and RASGRP2 are highly correlated with MEF2C expression; whereas levels of RAD51, PRKAA2, HRASLS2 correlate with MNDA. ROCK2 is correlated with SMAD3. Finally expression of RAD52 is associated both with those of MEF2C and SMAD3.

Transcriptional deregulation also occurred in genes participating in metabolic pathways, for instance, Figure 8 depicts the cholesterol biosynthesis pathway, again genes marked with a red star are abnormally expressed. Cholesterol biosynthesis and in particular the so-called Mevalonate pathway is a central process in the metabolic functioning: the starting point is Acetyl CoA which is a product of the metabolism of namely any source of energy -being carbohydrates, fats or proteins-. In the mevalonate branch of the cholesterol biosynthesis pathway (depicted in Figure 8), HMGCS1 and HMGCR proteins are enzymatically involved in the synthesis of mevalonate. Our analysis has shown that both genes HMGCS1 and HMGCR are abnormally regulated at the transcriptional level. Also in the reaction from 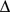-isopentyl 5 pyrophosphate to trans-trans farnesyl pyrophosphate an auxiliary enzyme GGPS1 is abnormally expressed. In the Lathosterol synthesis both TM7SF2 and NSDHL present affected mRNA expression levels. Expression levels of HMGCS1, HMGCR, GGPS1, NSDHL, TM7SF2 are all correlated with MEF2C expression in our analysis. It is noticeable that deregulation of the mevalonate pathway -and in particular of HMGCR - has been recently correlated with primary breast carcinoma [52]. Hypotheses relating metabolic dysfunction of the mevalonate pathway with primary breast cancer is also supported by the effect that cholesterol-controlling drugs have in patients with breast cancer [53], [54].

Further insight in the mechanisms that connect transcriptional deregulation in cancer and metabolic abnormalities could be found if we bring to attention a set of genes, consisting in PRKAA2, PRKAB2, CREB1, MAP3K1, DUSP4, TLR3, JUN, UBE2V1, TLR2 and MEF2C. All these genes turned out to be involved in the enrichment analyses both in the case of cancer-related pathways as well as metabolism-related pathways. PRKAA2 and PRKAB2 are AMPK subunits, CREB1 is a MAP kinase target and the rest are related with Toll-like receptor activity. Hence, kinase activity and signalling dysfunction seem likely to play a central role in cancer development, well beyond the usual RAS-ERK pathway. For more information, please refer to Table 4.

**Table 4:** Main REACTOME [28] pathways enriched in differentially expressed genes.

Un-adjusted probability	REACTOME Pathways	Gene ID
1.13E-03	Activation of ATR in response to replication stress	RFC2, RAD17, CDC25A,
		ORC5L, CDC6, CDC25C,
		MCM4
2.53E-03	Cholesterol biosynthesis	HMGCR, HMGCS1, TM7SF2,
		NSDHL, GGPS1
2.81E-03	G2/M Checkpoints	RFC2, RAD17, CDC25A,
		ORC5L, CDC6, CDC25C,
		MCM4
5.92E-03	Phospholipase C-gamma1 binds to the activated EGF receptor	PLCG1, EGFR
5.92E-03	EGFR activates PLC-gamma1 by phosphorylation	PLCG1, EGFR
5.92E-03	Active PLC-gamma1 dissociates from EGFR	PLCG1, EGFR
5.92E-03	EGFR interacts with phospholipase C-gamma	PLCG1, EGFR
1.48E-02	Myosin regulatory light chain phosphorylation by ROCK	ROCK1, ROCK2, MYH10
1.86E-02	PLC-gamma binds to the active receptor	PDGFRB, PLCG1
1.86E-02	PLC-gamma hydrolyses PIP2	PDGFRB, PLCG1
1.86E-02	ROCK activation by Rho	ROCK1, ROCK2
1.86E-02	CREB phosphorylation through the activation of Adenylate Cyclase	ADCY8, CREB1
2.07E-02	DNA Repair	POLB, APEX1, RFC2,
		POLR2F, POLR2I, BRCA1,
		FANCI, RAD51,RAD52,
		POLR2C
2.43E-02	MAPK targets/Nuclear events mediated by MAP kinases	DUSP4, JUN, CREB1,
		MEF2C
2.70E-02	Crk binds to the active PDGF receptor	PDGFRB, CRK
2.70E-02	LIM kinase phosphorylation by ROCK	ROCK1, ROCK2
2.77E-02	CREB phosphorylation through the activation of CaMKII	NEFL, GRIN1, CREB1
2.78E-02	Cell Cycle, Mitotic	BUB1B, RFC2, CEP250,
		AURKA, CDKN2A, ANAPC2,
		ORC5L, CENPF, TYMS,
		MCM4, PSMD14,CKS1B,
		DNA2, GMNN, CCNE2,
		CDC25C, CDC6, ODF2,
		CDC25A, ERCC6L, GORASP1
3.15E-02	E2F mediated regulation of DNA replication	CDC25A, ORC5L, CDC6,
		TYMS
3.67E-02	Interaction of MyoD-E protein with MEF2	MYOD1, MEF2C

### Gene Set Enrichment Analysis

Previous analysis in this work were mostly derived from the list of differentially expressed genes i.e. single-gene analysis. In order to include the collective behavior of genes within pathways and functional modules, we performed Gene Set Enrichment Analysis (GSEA) to determine whether members of gene sets 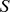 tend to occur at the top or bottom within a ranked list 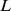 (genes showing largest difference between phenotypes) [55], [56]. GSEA was then applied to our expression data set (1191 samples) considering two sub-collections: Canonical Pathways (CP) and Cancer Modules (CM). Relevant parameters used for both sub-collections were the following: *permutations* - 1000, *scoring scheme* - weighted and *metric* - Signal2Noise.

Among the resulting statistically significant enriched gene sets (Nominal p-value 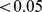 and FDR q-value 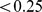), we obtain 77 pathways and 38 modules from CP and CM respectively. Table 5 shows some selected modules and pathways up-regulated in cancer that also involve genes with metabolic activity. These selected over-represented gene sets support our results from previous section in which we found deregulation of other genes beyond oncogenes and tumor suppressor genes (i.e. metabolic genes) promoting changes at the transcriptional level.

**Table 5:** Gene sets from the Molecular Signatures Database (MSigDB) corresponding to 2 of the 5 major collections [56].

Collection	No. gene	Enriched sets	Nominal	FDR	NES
	sets		p-value	q-value	
C2 Curated	3272				
Canonical Pathways	880	mTOR pathway	0.0	0.191	1.72
		Rho pathway	0.004	0.197	1.74
		Integration of energy metabolism	0.008	0.219	1.783
		Metabolism of proteins	0.0	0.188	1.809
C4 Computational	881				
Cancer Modules	454	module 159	0.002	0.148	1.61
		module 273	0.002	0.158	1.62
		module 346	0.013	0.177	1.67

Summary of the the GSEA results: some of the enriched gene sets involved in cancer and metabolism are shown (Nom. pval 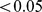 and FDR 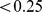).

**Figure 9:**
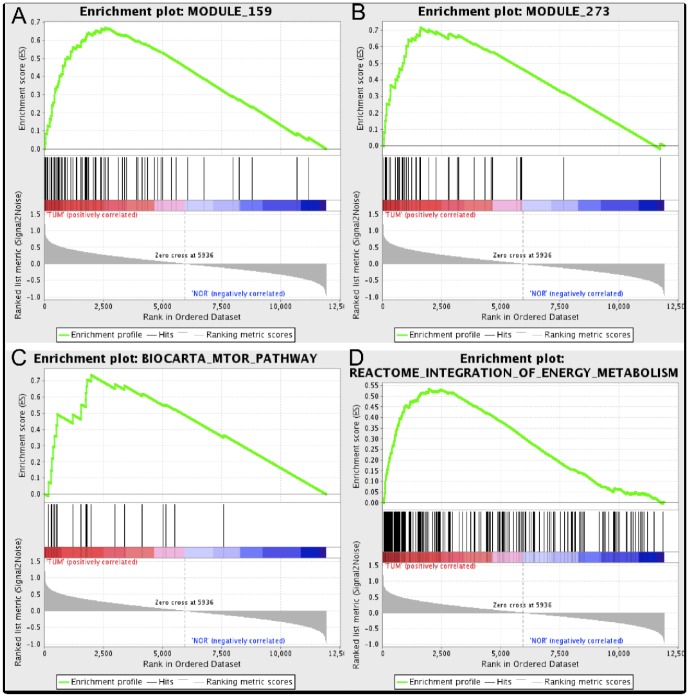
Enrichment score behavior. Show the distribution of four gene sets from the: A,B) Cancer Modules and C,D) Canonical Pathways; all of them were found up-regulated in cancer samples. Statistical significance of the shown plots can be found in Table 5.

Enrichment plots for selected cancer modules, in particular modules 159 (Nom. p-value = 0.002 and FDR = 0.148) and 273 (Nom. p-value = 0.002 and FDR = 0.158) are shown in Figure 9-A, B. Additionally, Figure 9-C, D show enrichment plots for cancer-metabolism related pathways i.e. mTOR (Nom. p-value
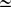
0.0 and FDR = 0.191) and integration of energy metabolism (Nom. p-value = 0.008 and FDR = 0.219) pathways. Within module 159, isoforms members from the RAS oncogene family (RAB10, RAB11A, RAB13, RAN, etc.) were found, additionally, genes CDC42 (not shown) and RHOA including in DNA damage (ATP dependent), Rho pathway and cell cycle progression (see Figure 7). Module 273 includes genes from the ATP synthase family, related to metabolic pathways, oxidative phosphorylation, CBFB and FOXO4 genes that participate in the regulation of nuclear SMAD2/3 signaling, MEF2C and POLR2 isoforms in the DNA repair pathway dependent on ATM. In module 346, ARSA, ASAH1, B4GALNT1 genes involved in metabolism of lipids and lipoproteins, B4GALNT1, phospholipase members of families A2, C and D (participates in mTOR pathway) were found.

### Core Gene Validation

Since the present work is a theoretical-computational analysis based on several sources for experimental data, no wet-lab validation *on biological samples* could be done, due to lack of sample-availability. However, an indirect validation procedure is performed by means of data mining for experimental results in the literature supporting our findings. For instance, *MEF2C* was identified as a candidate gene/molecular marker for the transition from ductal carcinoma *in situ* (DCIS) to invasive ductal carcinoma (IDC) and evaluated by RT-PCR. In reference [57], patient-matched DCIS/IDC samples were used to test expression profiling in Affymetrix oligonucleotide microarrays (GeneChip HG U133A and HG U133 plus 2.0). *MEF2C* was shown to be up-regulated in IDC compared with DCIS (p-value
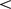
0.01 and FDR 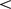
0.01). The average fold change for MEF2C in IDC vs DCIS was 2.03
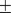
1.41 (see Table 6). In order to validate the previously mentioned result, real time PCR was performed in tumor specimens T796, T661, T787 and T808 showing also up-regulation of *MEF2C* and *in situ* hybridization was done to confirm its cellular specificity. *in situ* hybridization showed that *MEF2C* is present in the cytoplasm of DCIS and IDC, indicating its expression in epithelial tumor cells [57].

**Table 6:** Gene expression validation for *MEF2C* and *MNDA* in breast cancer samples.

Gene symbol	Fold change from microarray data	Fold change from real-time PCR	Experiment(reference)
*MEF2C*	2.03	T796 (1.69); T661 (1.79)	[57]
		T787 (2.89); T808 (1.38)	
*MNDA*	S1 (1.74); S2 (1.93)		[59]
	S3 (2.67); S4 (3.12)	*NB	

S1–S4 refer to different samples in microarray analysis, T796, T661, T787 and T808 refer to different tumor samples in RT-PCR.

* NB refers to experimental determination by Northern blot instead of RT-PCR.

Additionally, in ref. [58], protein expression of *MEF2C* in breast cancer cell lines was examined. Cellular lysates from exponentially growing MCF-10A, HMEC, MCF-7, T47D, ZR-75-1, and SKBR3 cells were analyzed by Western blot with antibodies specific to *MEF2C*. *MEF2C* expression in normal mammary epithelial cells and all breast cancer cell lines examined was observed. Results from this work suggest that Brk inputs to *p38* MAPK-dependent activation of MEF2 transcription factors in breast cancer cells.

In a study of differential expression in human breast epithelial cell lines irradiated with low doses of high linear energy transfer radiation, and treated with estrogen assessed with cDNA expression arrays, activity of *MNDA* was analyzed. *MNDA* showed a high level of altered expression (see table 6) and it was confirmed by gene-specific semiquantitative reverse transcription polymerase chain reaction, followed by Northern blot analysis. The results showed that the mRNA expression patterns for *MNDA* was consistent with the expression pattern seen on the array. *MNDA* showed upregulation in the transformed and tumorigenic cell lines compared a control (MCF-10F cell line) [59].

Expression of *MEF2C*, *SMAD3* and *POU2AF1* can be found as deregulated within two different platforms (HGU133A & HGU133plus2) in the BarCode database [60] for breast epithelium, stroma and lobular tissues. As can be seen in Table 7, *MEF2C* was differentially expressed in more than 60% of breast epithelium tumors samples (HGU133A) and more than 95% of breast stroma tumors samples (HGU133plus2). *MNDA* expression in breast carcinomas was examined on the EMBL-EBI database. Results from two experiments are shown in Table 8; *MNDA* was reported up-regulated in both studies.

**Table 7:** Barcode validation results for differential over-expression of *MEF2C*, *SMAD3* and *POU2AF1* within two platforms for several gene-probes in different breast cancer tissues [60], [78].

Gene symbol/Affy ID	Array	Tissue	% samples
MEF2C/209200_at	HGU133A	breast epithelium tumor	60%
MEF2C/209199_s_at	HGU133plus2	breast stroma tumor	95%
MEF2C/209199_s_at	HGU133plus2	breast tumor	60%
MEF2C/209200_at	HGU133plus2	breast tumor	60%
SMAD3/218284_at	HGU133plus2	breast lobular tumor	60%
POU2AF1/205267_at	HGU133A	breast epithelium tumor	60%

**Table 8:** MNDA expression status in two different microarray experiments also validate our finding [81].

Gene symbol/Affy ID	Experimental factor	Expression status	p-value	Experiment (reference)
MNDA/204959_at	breast carcinoma	up	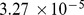	[79]
MNDA/204959_at	breast carcinoma	up	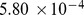	[80]

## Discussion

We have found an interesting trend in the transcriptional activity coefficients for and other well characterized gene probes. It could be seen [19] that the values of the chemical potentials of transcription 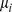 are in general lower for genes that act as transcription factors (such as *MNDA*, *POU2AF1*, *MEF2C* and *SMAD3*) and high for genes with no-known TF-activity (such as, for example, *IL2RB*, *CD69*, *TNFRSF1B* and *TNFRSF14*). One possible exception in the group of genes studied is *GLDC* which codes for a glycine-dehydrogenase enzyme that is anchored to the mitochondrion and has no reported evidence of transcription factor activity yet is grouped with the TF genes in the set of low chemical potential. This fact supports the TF
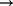
low 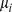 hypothesis. The physicochemical meaning of this finding seem to point-out to transcription factors as genes whose expression is regulated by lower activation-energy barriers. Since TF's are involved in the transcriptional activation of other genes, it is expected that they are synthesized first when energy is started to being released by metabolic processes in the cell. Transcriptional targets should, in general be synthesized later and with higher activation energies. Even between transcription factors, we hypothesize that genes upstream in the transcriptional cascade must in general present lower chemical potentials of transcription. This mechanism would function thus as a *lock-in-the-trigger* for transcriptional cascading. It is important to stress that the chemical potentials 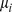 for *spontaneous* transcription do not show a big difference between TFs and Target Genes (TGs) at low gene expression intensity levels (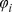) but show a significant difference at high values of 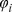 (recall that the values of 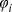 at which the chemical potential becomes negative are the saturation constants 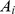 and these show statistical significant differences between TFs and TGs). Thus, higher saturation limits for the chemical potentials of TFs suggest both stability and spontaneity in the expression of these as compared to TGs. Pathway analysis also make evident the fact that transcriptional deregulation occurs not only at the single-gene level, since in some cases several genes in the same pathway are affected.

With regards to more particular findings, we have already discussed that deregulation seem to be present at the pathway level. Specifically we have found two related sets of pathways affected by deregulation in several of their genes. These two sets correspond grossly with cancer-related pathways (cell cycle, DNA repair, apoptosis) and metabolic control pathways (cholesterol synthesis, AMPK release, etc.). Some affected genes are actually part of both kinds of pathways. These genes are correlated with MEF2C expression (Figure 6). As we mentioned, the network structure in Figure 6 suggest some kind of control between MEF2C and MNDA, however since our inference method is based in (nonlinear) correlations, causality (i.e. directionality of the transcriptional regulation interactions) could not be ascribed to either of the genes. Bayesian and other inference approaches could be useful for that means, but they would require actual time-course experiments in many, many samples to attain statistical significance. In the case of human samples, for logistic and financial reasons, this does not seem at hand in the near future. A thermodynamic analysis -additional to the correlation network study- such as the one we presented, supplies us with an alternative, that although it involves some assumptions, could give us some hints on the causal structure. It should be stressed however, that these clues should be considered hypotheses to be experimentally verified or disregarded, either on the light of more detailed experiments or more specific thermodynamic assumptions and not conclusive results nor proven facts.

Let us recall the results of the thermodynamic analysis and the correlation network analyses. In Figure 3 we observed that whereas MEF2C presents comparatively low mRNA concentrations and low concentration variances, along with a very high connectivity (degree), MNDA presents both a high concentration and high variability in the sample-set, as well as the second highest degree. In the other hand, SMAD3 and POU2AF1 present medium-to-low concentrations, and much smaller connectivities. However, POU2AF1 presents a high variability, while SMAD3 has a medium-to-small variance. If we *assume* that, in effect, there is a kind of control loop between MNDA and MEF2C somehow fine-tuned by the action of POU2AF1 and SMAD3 (and their interactors); then we may sketch a possible causal scenario if we consider that energy is been released gradually within the cells. MNDA is present at high concentrations (Figure 3) almost always because its associated chemical potential (i.e. free energy of formation) is low (Figure 5) and hence it is easier to synthesize, in the other hand MEF2C is present in small amounts (Figure 3) because its chemical potential is high (Figure 5) so it is more difficult to synthesize. This seem to suggest that we have a *balance* between the concentrations of MNDA and MEF2C. If energy release within the tumor cells occurs as a consequence of gradual activated processes, then MNDA should be transcribed first and MEF2C expression may be regulated by MNDA and its interactors. Once MEF2C is being produced, then activation of its many transcription targets should cause abnormal regulation in the already mentioned cancer and metabolic related pathways, thus causing diseased cellular states and, ultimately, carcinogenesis.

With regards to the design of possible experimental protocols to verify some of these findings, we must notice that experimental techniques in genomics are rapidly evolving, in such a way that probing the cell in real time under almost *in vivo* conditions is now becoming possible. In particular with regards to experimental verification of our findings, there have been several instances in which related work has been done. One approach to provide real-time semi-quantitative analysis of transcription is the imaging of reporter gene expression, for example, using firefly luciferase [61], [62]. There is also the need for protein production/degradation rates to experimentally assess gene expression dynamics [63]. Nevertheless, in the most successful cases it has been even possible to account for fluctuations and stochastic components in the dynamics of gene expression [64]. Other modern techniques to monitor the dynamics of gene expression are based in quantitative measurements of polymerase chain reaction (q-PCR) often used in conjunction with immunoprecipitation [65], [66]. In the near future it is also very likely that techniques such as microcalorimetry at a single cell level, and especially Isothermal Titration Calorimetry (ITC) could be applied on a real-time basis to monitor changes in local thermodynamics within the cell [67]. These experiments will also shed light in the thermal component of messenger RNA dynamics, and thus will serve to fine-tune the predictions of the model presented here.

### Conclusions

In this paper, we have analyzed the role that thermodynamic fluctuations in energy at the cell-level play in the synthesis of transcription factors *MNDA*, *POU2AF1*, *MEF2C* and *SMAD3* and how can this energetic constrains be related with the presence of primary breast carcinomas. In doing so we studied systematically mRNA levels for high throughput, whole-genome gene expression profiling. A set of 1191 publicly available microarrays was first studied by inferring gene regulatory networks based in a non-linear correlation measure between gene expression vectors arranged in a surrogate dynamic model of tumor progression. In the other hand, a non-equilibrium thermodynamic formalism was used to calculate the concentration-dependent gene expression intensity, the chemical potentials of transcription and their associated affinities in order to establish energetic constraints that help us to evaluate the biological hypotheses. Hence, a connection was established between mRNA concentration patterns -as given by experimental gene expression profiles- and local cell energetics - by means of these irreversible thermodynamical quantities. By analyzing the different patterns of gene expression for the selected genes, the corresponding non-equilibrium energetics, as well as their correlation structure as given by network analysis, an integrative model for the action of a core set of master regulator genes was developed. We analyzed in the surrogate *dynamic* model already mentioned to look up for transcriptional regulation paths. All these analyses suggest a novel potential role of transcription factor energetics in tumor development.

In particular, by using a data integration approach that combines experimental evidence for high throughput genome wide gene expression, a non-equilibrium thermodynamics analysis, nonlinear correlation networks as well as database mining, we were able to hypothesize about the role that transcription factors MEF2C and MNDA may have as *master regulators* in primary breast cancer phenomenology, as well as the possible interrelationship between malignancy and metabolic dysfunction. Nevertheless, we can never emphasize enough that these findings should be regarded as hypothesis generators rather than as conclusive results. However, we believe that systematic studies relying in data integration guided by well-founded physical principles rather than intuition may become mandatory in time. This work intends to point-out in such direction. However, further, deeper investigations are needed in this direction in order that our understanding of these extremely complex phenomena will be substantially increased.
